# Role of neuroinflammation in neurodegeneration: new insights

**DOI:** 10.1186/s13195-017-0241-2

**Published:** 2017-03-04

**Authors:** Róisín M. McManus, Michael T. Heneka

**Affiliations:** 1 German Center for Neurodegenerative Diseases (DZNE), Sigmund Freud Str. 27, 53127 Bonn, Germany; 2 Department of Neurodegenerative Disease and Gerontopsychiatry/Neurology, University of Bonn Medical Center, Sigmund-Freud Str. 25, 53127 Bonn, Germany

**Keywords:** Alzheimer’s disease, Amyloid-β, Infection, Neuroinflammation, Aging, T cells

## Abstract

Previously, the contribution of peripheral infection to cognitive decline was largely overlooked however, the past 15 years have established a key role for infectious pathogens in the progression of age-related neurodegeneration. It is now accepted that the immune privilege of the brain is not absolute, and that cells of the central nervous system are sensitive to both the inflammatory events occurring in the periphery and to the infiltration of peripheral immune cells. This is particularly relevant for the progression of Alzheimer’s disease, in which it has been demonstrated that patients are more vulnerable to infection-related cognitive changes. This can occur from typical infectious challenges such as respiratory tract infections, although a number of specific viral, bacterial, and fungal pathogens have also been associated with the development of the disease. To date, it is not clear whether these microorganisms are directly related to Alzheimer’s disease progression or if they are opportune pathogens that easily colonize those with dementia and exacerbate the ongoing inflammation observed in these individuals. This review will discuss the impact of each of these challenges, and examine the changes known to occur with age in the peripheral immune system, which may contribute to the age-related vulnerability to infection-induced cognitive decline.

## Background

It has been estimated that by the year 2050 the population of individuals over the age of 60 will double from 901 million in 2015 to 2.1 billion people worldwide [1]. Importantly this increase in life expectancy will go hand-in-hand with an increase in age-related diseases, with the elderly currently expected to spend more of their later years in overall ill-health [2]. Indeed dementia, one of the principle causes of disability in the elderly, currently affects 44 million people globally, with this figure expected to increase to over 135 million people by the year 2050 [3]. As the annual cost of dementia care is expected to increase from $600 billion to $1 trillion over the next 15 years [3], finding a way to prevent disease progression is vital. This review will summarize the impact of inflammation on the progression of neurodegeneration, with a focus on the role of infection-related neuroinflammation in dementia, which is a rapidly growing area of interest in the field.

## Review

### Infection in the elderly and in Alzheimer’s disease

The immune system undergoes many changes with age that leaves the elderly more susceptible to infection [4], indeed older individuals are more vulnerable to bacterial or viral infections of the urinary or respiratory tract, with influenza-related morbidity also increased in this group [5, 6]. Sepsis, which is caused by severe infection, can also lead to permanent cognitive dysfunction, particularly in older individuals [7]. Importantly, infectious burden in the elderly is associated with mini-mental state examination (MMSE) scores below 24, which indicate dementia [8]. This is in line with a previous study that linked infection with lower MMSE scores, however Hodgson and colleagues also observed that 36% of elderly subjects with dementia had an infection which was formerly undiagnosed [9]. Unfortunately, the symptoms of infection often present atypically in this group [10] and, as dementia patients are often unable to communicate their symptoms [11], diagnosis is difficult. To further complicate matters, bacterial resistance is often increased in older patients [12].

Individuals with Alzheimer’s disease (AD) are even more vulnerable to the effects of peripheral infection. In a 10-year follow-up study, delirium (which is often caused by infection) correlated with an eightfold increase in dementia development [13]. Furthermore, the cognitive capabilities of AD patients worsened significantly after an episode of delirium, which has been confirmed by others [14]. Indeed poor health [15] and viral burden [16] have been linked with cognitive impairment and AD development in the elderly. Natalwala and colleagues found that the incidence of many infectious conditions such as pneumonia, lower respiratory tract, or urinary tract infections is higher in AD patients than healthy, age-matched controls [17]. Previous studies have demonstrated that numerous infections over a 4-year period doubled the risk of AD development [18]. Indeed cognitive decline has been observed just 2 or 6 months after a resolved peripheral infection, with an association between cognitive impairment and circulating proinflammatory cytokines [19, 20]. Pneumonia is a frequent, if not the most common, cause of death in AD [21, 22], conversely, vaccination against influenza and other infections significantly reduced the risk of AD development [23, 24] and antibiotic treatment has been observed to slow cognitive decline in patients [25]. Many specific viral, bacterial, and fungal pathogens are suspected to play a role in the progression of neurodegeneration including herpes simplex virus type 1 (HSV-1), *Chlamydia pneumonia,* spirochetes, and *Candida* [26–28] thus, the contribution of each pathogenic group will be examined in detail.

### Viral infections

Chronic infection with HSV-1 and cytomegalovirus (CMV) has been implicated in neurodegeneration. HSV-1 is typically a lifelong, latent infection of the central nervous system (CNS) and, while the virus has been found in the brains of control and AD subjects, viral deoxyribonucleic acid (DNA) was located in regions such as the hippocampus, which are particularly affected in AD [29]. HSV-1 is a risk factor for AD in people carrying the apolipoprotein E epsilon 4 (*APOE4)* allele, indeed the allele frequency is much higher in the HSV-1-infected than non-infected AD population [28, 30]. In vitro studies have demonstrated that HSV-1 triggers amyloid precursor protein (APP) processing, resulting in the production of amyloid β (Aβ) via β- and γ-secretases [31], and murine studies have shown that *APOE4*-expressing mice have a significantly enhanced viral burden after infection with HSV-1 [32]. It is believed that HSV-1 outcompetes ApoE4 in binding to heparan sulfate proteoglycans (HSPG) on the cell surface, thus facilitating viral internalization and infection of the host cell in *APOE4* carriers in particular [26].

CMV is another lifelong, latent infection that, along with HSV-1, was associated with lower MMSE scores in the elderly [16]. In a 5-year follow-up study, CMV was linked with faster cognitive decline and development of AD [33], which supports two other reports that found an association between CMV seropositivity and AD development [34, 35]. Interestingly, Westman and colleagues observed that the peripheral blood mononuclear cells (PBMCs) from CMV^+^ AD patients were more reactive after stimulation than non-infected patients, suggesting that CMV is an inflammatory promoter in AD [36].

### Bacterial infections

A number of bacterial pathogens have also been associated with the development of AD. *Chlamydia pneumonia* is an obligate intracellular, Gram-negative bacteria that was first observed in the postmortem AD brain by Balin and colleagues in 1998 [37], although the finding has been replicated many times since [38, 39]. Infection with *C. pneumonia* is associated with a fivefold increase in AD development [40], and AD patients have increased levels of *C. pneumonia*-specific antibodies in circulation in comparison with control subjects [34]. It is believed that the bacteria can cross the blood-brain barrier (BBB) via the olfactory route or within infected monocytes [41]. Once inside the CNS, *C. pneumonia* can infect microglia, astrocytes, and neurons. Importantly, Gérard and colleagues observed infected cells containing viable, metabolically active pathogens in close proximity to AD-plaque pathology [39]. Similar to HSV-1, AD patients with the *APOE4* allele are more susceptible to infection with *C. pneumonia* as a significantly greater bacterial burden was observed in regions such as the hippocampus in comparison to those without *APOE4* [38]. *C. pneumonia* can inhibit neuronal apoptosis in vitro, thus facilitating the maintenance of a chronic infection [42]. Interestingly, intranasal infection of mice with *C. pneumonia* induced Aβ deposition in the brain, which co-localized with reactive glia [43], importantly *C. pneumonia* remains active in the murine CNS for months after infection [44].

*Heliobacter pylori* is a Gram-negative bacteria that grows in the digestive tract and was recently demonstrated to have a significant association with the development of dementia [45]. In older individuals, the presence of *H. pylori* IgG antibodies was associated with decreased cognitive performance [46], indeed research has shown that AD patients also have increased *H. pylori* seropositivity in the serum and cerebrospinal fluid (CSF) [47]. Furthermore, Kountouras and colleagues have demonstrated that individuals with AD had an increased incidence of *H. pylori* infection of the gastric mucosa compared to controls [48]. Within AD patients, those infected with *H. pylori* had more severe dementia, characterized by lower MMSE scores, with increased proinflammatory cytokines and tau levels in the CSF [49]. A 20-year follow-up study also found *H. pylori* to be a significant risk factor for the development of dementia, although even at baseline those positive for *H. pylori* had lower MMSE scores [50]. It has been reported that elimination of *H. pylori* infection reduced the mortality rate of AD patients when examined 5 years later [51]. Furthermore, AD patients who were treated for their infection and remained *H. pylori*-free for 2 years had improved cognition than when they were first examined, while those who were still positive for the bacterium had further declined [52]. Animal studies have demonstrated that intraperitoneal injection of *H. pylori* filtrate into rats increased the concentration of Aβ_42_ in the cortex and hippocampus, which was associated with memory deficits and impaired spatial learning [53]. This group also reported that *H. pylori* filtrate significantly increased tau phosphorylation in neuronal cultures in vitro and in the rat hippocampus in vivo [54]. Conversely, infection of C57BL/6 J mice with *H. pylori* did not affect amyloid deposition when assessed 18 months later [55], however the effect of *H. pylori* on amyloid pathology in AD-transgenic mice has yet to be examined.

Periodontitis is another a risk factor for AD and it has been demonstrated that healthy elderly individuals with periodontal disease have a higher accumulation of amyloid in the CNS [56], with an association found between elevated interleukin (IL)-6 and tumor necrosis factor (TNF)α in the circulation and periodontitis in AD patients [57]. A common cause of periodontitis is spirochete infection, which is a Gram-negative, neurotropic bacterium. The periodontal spirochete pathogen *Treponema* has been detected in the AD brain, with co-infection of multiple *Treponema* species observed in some patients [58]. Many other species of periodontal pathogens have been found in the AD brain including lipopolysaccharide (LPS) from *P. gingivalis* [59] and *Borrelia burgdorferi* [58, 60, 61]. Importantly, *B. burgdorferi* co-localised with Aβ deposits in patients [61] and was found to induce Aβ deposition by glial and neuronal cells in vitro [62]. Indeed, it has recently been suggested that bacterial amyloid, along with host-derived Aβ, are constituents of the senile plaques observed in AD [63]. A number of studies have found that significantly more AD patients have a spirochete infection when examined in the post-mortem brain, than controls [58, 60]. In addition, AD patients have increased levels of *B. burgdorferi*-specific antibodies in circulation [34], and a recent study demonstrated a tenfold increase in the occurrence of AD with spirochete infection [40].

### Fungal infections

There have been a number of reports over the past 3 years on the contribution of fungal infections to the progression of AD. In 2014, Alonso and colleagues first demonstrated the presence of fungal proteins and DNA in the AD brain [64]. Many different species were detected including *Saccharomyces cerevisiae*, *Malassezia globosa*, *Malassezia restricta*, and *Penicillium*. Analysis of CSF samples from patients also revealed the presence of *S. cerevisiae*, *M. globose*, and *M. restricta* DNA, while the CSF levels of *Candida albicans* and *C. glabrata* proteins were significantly greater in those with AD [65]. In both studies it was observed that many AD patients were co-infected with a number of fungal species, while no fungal DNA was detected in control samples. In line with this finding, AD patients also have greater seropositivity to *C. albicans* and *C. glabrata* [66]. Immunohistochemical analysis identified fungal material inside neuronal cells in the postmortem AD brain, including macromolecules from *Candida glabrata*, *Penicillium notatum*, and *C. albicans* [67]. Furthermore, this group have found fungal material both intra- and extra-cellularly, and in many brain regions including the frontal cortex, hippocampus, and the blood vessels of the CNS, with mixed-fungal infections observed in multiple patients [27]. It has yet to be established whether the fungal infection co-localises with Aβ or if the infection has a direct or indirect effect on amyloid production in the CNS.

Interestingly, it has been suggested that Aβ may also function as an antimicrobial peptide (AMP). In vitro studies have confirmed that Aβ has antimicrobial activity against a range of pathogens and was as effective, or even more potent, than LL-37 which is an established human AMP [68]. Importantly, *C. albicans* was the microbe most sensitive to synthetic Aβ, and brain homogenates from AD patients, but not controls, were also capable of inhibiting fungal growth. It was recently demonstrated that Aβ protects against *C. albicans* infection in glial cells in vitro and in nematodes in vivo [69]. In addition, Aβ inhibits HSV-1 viral replication in vitro, and protects mice from *Salmonella* Typhimurium infection in vivo, which led the authors of both studies to conclude that Aβ may have a previously unknown protective role in innate immunity, along with the pathogenic characteristics that are extremely well studied [69, 70].

### Why are the elderly more susceptible to these infections?

The emerging evidence strongly indicates that infection has a significant role in the development of, and progression to, dementia, with a growing list of pathogens specifically associated with AD or Aβ deposition. This may be due in part to some of the changes that are known to occur to the immune system with age. One of the key changes in the adaptive immune system is the involution of the thymus, resulting in a dramatic decrease in the production of new T cells [71]. With age, there is an overall decrease in naive T cells, and a corresponding increase in memory T cells [72]. This is associated with a reduction in naive T cell diversity after the age of 65 [71], with clonally expanded subsets of memory T cells often observed in this age group, which can occur from chronic or repeat infections [4, 72]. Together, this can limit the capacity of the individual to induce a sufficient immune response to new infectious challenges. In adults, the remaining pool of naive T cells is maintained via proliferation [73], however over their life-span, these naive cells can be exposed to stressors such as oxidative species or changes in the availability of cell-survival factors, which can affect their function [74].

Interestingly, many studies have demonstrated that T cell activation is even further altered in AD. The population of naive T cells is significantly decreased in AD patients, with an increase in memory T cells in comparison with age-matched, healthy controls [75–77]. In addition, it has been reported that AD patients have T cells with shorter telomeres, and telomere length significantly correlated with AD severity [78]. AD patients have increased T cell reactivity to Aβ [79, 80], and the phenotype of the T cells in circulation is shifted, with increased CD4^+^IFN-γ^+^ and CD8^+^IFN-γ^+^ T cells observed [81, 82]. Saresella and colleagues also reported an Aβ-specific population of Th17 and Th9 cells that was increased in AD patients in comparison to healthy control subjects [75].

These changes can have a critical impact on the CNS, as activated T cells have been found in the CSF of AD patients [83, 84] and these cells have been reported in the human brain, with greater numbers reported in the brains of AD patients [85, 86]. Importantly, T cells were found in close association with microglia [86], which are known to have an antigen-presenting phenotype in AD [85, 87, 88]. Animal studies have demonstrated the presence of IFN-γ^+^ Th1 cells and IL-17^+^ Th17 cells in the CNS of aged APP/PS1 mice [89, 90]. In addition, respiratory infection can have a significant effect on the phenotype of T cells in the CNS [90]. It was observed that peripheral infection increased the deposition of Aβ in the brain, which was associated with increased T cell infiltration and microglial activation in older, but not younger, APP/PS1 mice.

It has also been reported that the innate immune system undergoes changes with age and in AD. Monocytes prepared from individuals with discrepant memory IQ had increased expression of CD11b, Toll-like receptor (TLR)2, and TLR4 [91]. Indeed the population of circulating myeloid dendritic cells (DCs) is decreased in the elderly [92] and these cells are further reduced in AD [93]. AD patients had increased levels of ICAM-1^+^ monocyte-derived DCs [94] and increased expression of MHC class II and CD16 on CD14^+^ monocytes [95]. Saresella and colleagues also found a significant increase in IL-6- and IL-23-producing CD14^+^ monocyte/macrophages in AD patients, while IL-10^+^ cells were reduced [75]. Furthermore, it was recently demonstrated that circulating NLRP3^+^caspase 1^+^ and NLRP3^+^caspase 8^+^ monocytes are increased in AD, and these cells produced significantly greater levels of IL-1β and IL-18 after LPS and Aβ treatment [96]. This is in line with our previous work demonstrating an important role for the NLRP3 and caspase-1 pathway in the progression of AD pathology, both in AD patients and APP/PS1 mice [97]. Myeloid cells from memory-impaired individuals also have a greater stimulus-induced proinflammatory response [91], which mirrors animal studies demonstrating the same effect in bone marrow-derived macrophages from APP/PS1 mice [98]. However, it has been found that DCs from AD patients have a reduced ability to stimulate T cell proliferation [94]. The innate immune system provides the first line of defense against infectious agents, thus an altered response here can have severe consequences for the individual and their ability to control, and respond to, infection. As peripheral myeloid cells have been detected in the AD brain [99], changes in the phenotype of these circulating cells suggest that those which have infiltrated the CNS are similarly altered.

## Conclusion

**Fig. 1:**
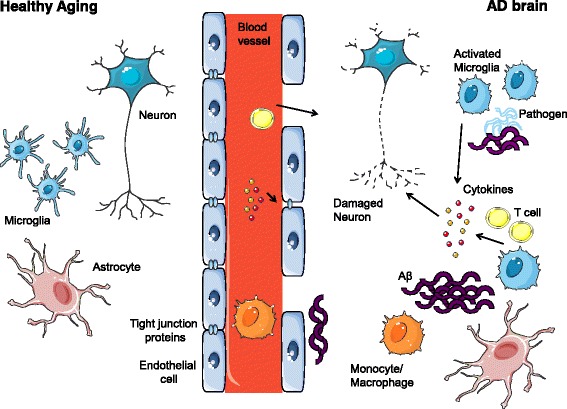
The impact of infection on Alzheimer’s disease pathology. Healthy aging is accompanied by increased blood-brain barrier (BBB) permeability and an elevation in baseline inflammation however, Alzheimer’s disease (AD) is characterized by a significant increase in microglia activation, amyloid β (Aβ) deposition, BBB disruption and neuronal loss, far beyond that observed in non-demented, age-matched controls. It is established that microglia produce a range of cytokines in response to the growing presence of Aβ, however, with the increased number of T cells in the AD brain that have the capacity to interact with microglia, this can lead to elevated cytokine production. In addition, pathogens have been found in the AD brain, indeed many species were in close association with Aβ plaques. The presence of these microorganisms can exacerbate the ongoing neuroinflammation, thus negatively affecting nearby neuronal and glial cells leading to neurodegeneration. The situation is further confounded by inflammatory events occurring in the periphery, such as respiratory infection. This can result in increased immune cells and cytokines in circulation, which would have little influence on the healthy CNS, but the inflamed AD brain cannot efficiently handle this extra challenge. As the innate and adaptive immune cells of AD patients have altered reactivity, together they have the potential to further affect the BBB and exacerbate inflammatory changes within the CNS. This is an original figure, which was designed for this manuscript

It is clear from the evidence that AD patients are more vulnerable to the effects of peripheral infection than their age-matched, healthy counterparts. Importantly, it is indisputable that many specific viral, bacterial, and fungal infections are associated with AD development, although whether these pathogens are a direct cause of dementia or instead are advantageous, infiltrating microorganisms that exacerbate the neuroinflammation already ongoing in these individuals remains to be confirmed. Importantly, the BBB of AD patients is significantly leakier than in healthy subjects, which facilitates infiltration of peripheral immune cells [100] and possibly these infectious pathogens (Fig. 1). Together, this review demonstrates the critical need for early detection and treatment of infections in the elderly and in those with dementia. As infectious diseases can present atypically in this group, frequent screening and vaccination are key to preventing infection-related deterioration of cognition until new therapies are established that can protect the elderly from these unnecessary insults.

## Abbreviations

AD: Alzheimer’s disease
AMP: Antimicrobial peptide
APOE4: Apolipoprotein E epsilon 4
APP: Amyloid precursor protein
Aβ: Amyloid β
BBB: Blood-brain barrier
CMV: Cytomegalovirus
CNS: Central nervous system
CSF: Cerebrospinal fluid
DC: Dendritic cell
DNA: Deoxyribonucleic acid
HSPG: Heparan sulfate proteoglycans
HSV-1: Herpes simplex virus type 1
IL: Interleukin
LPS: Lipopolysaccharide
MMSE: Mini-mental state examination
NLRP3: NOD-, LRR- and pyrin domain-containing 3
PBMC: Peripheral blood mononuclear cell
TLR: Toll-like receptor
TNF: Tumour necrosis factor
